# Statistically Optimized Polymeric Buccal Films of Eletriptan Hydrobromide and Itopride Hydrochloride: An In Vivo Pharmacokinetic Study

**DOI:** 10.3390/ph16111551

**Published:** 2023-11-02

**Authors:** Awaji Y. Safhi, Waqar Siddique, Muhammad Zaman, Rai Muhammad Sarfraz, Muhammad Shafeeq Ur Rahman, Asif Mahmood, Ahmad Salawi, Fahad Y. Sabei, Abdullah Alsalhi, Khalid Zoghebi

**Affiliations:** 1 Department of Pharmaceutics, College of Pharmacy, Jazan University, Jazan 45142, Saudi Arabia; asafhi@jazanu.edu.sa (A.Y.S.); fsabei@jazanu.edu.sa (F.Y.S.); aalsalhi@jazanu.edu.sa (A.A.); 2 Riphah Institute of Pharmaceutical Sciences, Riphah International University, Lahore Campus, Lahore 54000, Pakistan; 3 Faculty of Pharmaceutical Sciences, University of Central Punjab, Lahore 54590, Pakistan; 4 College of Pharmacy, University of Sargodha, Sargodha 40100, Pakistan; 5 Department of Pharmacy, University of Chakwal, Chakwal 48800, Pakistan; asif.mahmood@uoc.edu.pk; 6 Department of Pharmaceutical Chemistry, College of Pharmacy, Jazan University, Jazan 45142, Saudi Arabia; kzoghebi@jazanu.edu.sa

**Keywords:** buccal film, drug delivery, plasticizer, surfactant, immediate dosage form

## Abstract

A migraine is a condition of severe headaches, causing a disturbance in the daily life of the patient. The current studies were designed to develop immediate-release polymeric buccal films of Eletriptan Hydrobromide (EHBR) and Itopride Hydrochloride (ITHC) to improve their bioavailability and, hence, improve compliance with the patients of migraines and its associated symptoms. The prepared films were evaluated for various in vitro parameters, including surface morphology, mechanical strength, disintegration test (DT), total dissolving time (TDT), drug release and drug permeation, etc., and in vivo pharmacokinetic parameters, such as area under curve (AUC), mean residence time (MRT), half-life (t_1/2_), time to reach maximum concentration (T_max_), and time to reach maximum concentration (C_max_). The outcomes have indicated the successful preparation of the films, as SEM has confirmed the smooth surface and uniform distribution of drugs throughout the polymer matrix. The films were found to be mechanically stable as indicated by folding endurance studies. Furthermore, the optimized formulations showed a DT of 13 ± 1 s and TDT of 42.6 ± 0.75 s, indicating prompt disintegration as well as the dissolution of the films. Albino rabbits were used for in vivo pharmacokinetics, and the outcomes were evident of improved pharmacokinetics. The drug was found to rapidly permeate across the buccal mucosa, leading to increased bioavailability of the drug: C_max_ of 130 and 119 ng/mL of ITHC and EHBR, respectively, as compared to 96 (ITHC) and 90 ng/mL (EHBR) of oral solution. The conclusion can be drawn that possible reasons for the enhanced bioavailability could be the increased surface area in the form of buccal films, its rapid disintegration, and faster dissolution, which led toward the rapid absorption of the drug into the blood stream.

## 1. Introduction

A migraine is a condition of thrombotic pain, which leads to a disturbance in routine life. There are a lot of associated symptoms with migraines, where nausea and vomiting are most prevailing due to gastric stasis. Such symptoms also need to be addressed using anti-emetics as a remedy, but gastric stasis causes a decrease in the absorption of the drugs and, ultimately, the failure of this oral therapy. This fiasco results in a change in the route of administration, e.g., parenteral, which reduces the patient compliance. A large number of studies have reported that trans bucco-mucosal delivery of the drug could be an effective alternative to the oral and parenteral, which bypasses the absorption of the drug through trans-GIT. It not only helps to avoid the barrier caused by gastric stasis, but also improves the bioavailability of the drug by circumventing the first pass hepatic metabolism. Hence, a trans bucco-mucosal delivery of the drug in the form of buccal films would be an effective way to improve patient compliance.

The oral cavity is surrounded by lips from the anterior side, and facial arches are present on its posterior side just anterior to the tonsils and parallel to the cheeks. The superior side of the oral cavity has a palate, along with the muscular floor. The oral cavity, which is termed an oral vestibule, has a tongue, along with cheeks, teeth, and lips while the other part comprises teeth and a medial region. The mouth lining is covered with a mucous membrane, having a stratified squamous epithelium. The oral cavity is also moistened by salivary secretions from the salivary glands that are a thick mucus. The oral cavity acts as a gateway for the digestive tract and plays a vital role in the ingestion of essential nutrients [1].

Films are thin polymer dosage forms meant to be retained in the oral, sublingual, or tongue area. They adhere to the administration site and become hydrated by the presence of mucous membranes after the swelling and dissolution phenomena take place. Due to first-pass avoidance, the presence of a high blood supply, and administration, oral mucosal ease is a striking route for the administration of medications. As buccal films remain intact for a longer period, they exhibit a higher release and availability of drugs compared to other routes, such as suspensions or solutions. The release pattern in films depends on their mode of use or formulation design. These can be used for both local and systemic effects. They can be prepared in one or more layers as well as in a modified release format. They increase patient compliance due to their small size, flexibility, and increased absorption time.

The most common solvent evaporation method was used for the formulation of films; in this technique, all the ingredients, including the active drug, plasticizer, and polymer, are solubilized in water to make a solution. Then the ready solution was transferred to Petri dishes of the required size and allowed to cool or evaporate the water content [2,3,4,5]. To speed up the drying process, the prepared films were also placed in an oven at 40 °C. By carefully using a blade or scalper, the films were removed from Petri dishes and kept for 24 h in a desiccator. Finally, the prepared films were cut into the required sizes [3,6].

As migraines cause the population to be handicapped, it is ranked 19th among the general population and 12th among women. The World Health Organization (WHO), ranked migraines among the 20 top diseases worldwide, which affects about 15% [7] of the general population, out of which 17% are females and 6% are males [8]. Most migraineurs start developing migraines between the age group of 25–55 [7]. One in every eight citizens globally is affected by migraines [9]. Preventive and abortive strategies were used to treat the disease. The basic purpose of the treatment strategy is to relieve stress [10].

Triptans were regarded as serotonin (5-hydroxytryptamine, or 5-HT) agonists and bear extreme affinity for 5-HT1D and 5-HT1B receptors. It was suggested that triptans cause the vasoconstriction of cranial nerves and relieve migraine attacks with their action on 5-HTIB receptors in postsynaptic blood vessels and smooth muscles. However, recent studies revealed that triptans do block vasoactive peptides released from perivascular trigeminal neurons with their action on 5-HTID presynaptic receptors present at the terminal ends of nerves. Triptans that bind to presynaptic 5-HT1D receptors and appear at the dorsal point block the release of the neurotransmitter that will activate the thalamus due to the presence of second-order neurons approaching it. It also facilitates the inhibitory system from descending the pain [11].

**Figure 1:**
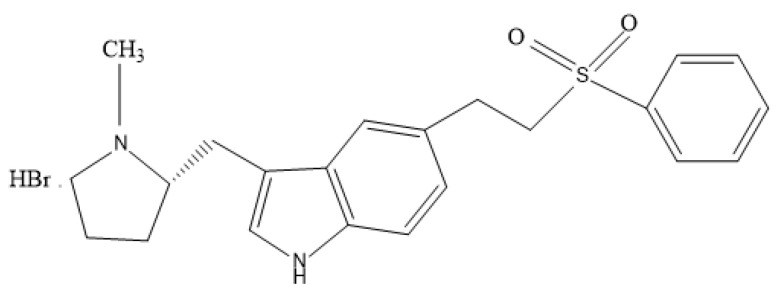
Chemical structure of EHBR.

Eletriptan hydrobromide (EHBR) (represented in Figure 1) belongs to the second generation of the triptan family, which was meant to be used to cure migraines [12]. On 26 December 2002, EHBR was accepted by the US FDA as an anti-migraine drug [13].

EHBR has a mode of action in which it reduces the swelling of blood vessels present in the brain [12].

EHBR binds on 5-5-HT1D, HT1B, [14], and 5-HT1F receptors in the cerebral blood vessels and perivascular nerve terminals [15]. Here, it causes vasoconstriction, decreases the release of neuropeptides at the central level, and hinders the path towards trigeminal nucleus caudalis, which blocks the pain transmission. Due to gastric stasis, the absorption of the drug is delayed in migraineurs [16,17,18]. The drug attains 50% bioavailability after oral administration [12,19,20,21]. After its oral administration, 85% of the drug binds with plasma protein [13]. Meanwhile, the half-life of the drug varies from 4.8 to 7.0 h after its oral administration [22,23]. EHBR belongs to BCS class II drugs.

Gastric stasis or gastroparesis was named as the delayed evacuation of stomach contents without any mechanical obstruction. Gastroparesis has some circumstances, such as nausea, weight loss, vomiting, and bloating [24,25]. An amount of 84% of patients experience vomiting, nausea is experienced in 92% of the patients, and 60% of the patients were exposed to early satiety during the exposure of gastroparesis [26]. During gastroparesis, normally, vomiting is induced due to the pain caused by the migraine, which causes a delayed absorption of the medication [24]. Migraine attacks cause delayed gastric emptying [27]. If the condition of gastroparesis persists for a long duration, it may result in esophagitis, malnutrition, electrolyte disturbance, and acute renal failure due to less volume [24]. The condition of gastroparesis was cured by giving medications that have prokinetic activity, such as 5-HT4 and dopaminergic agonists. Studies proved that the usage of non-oral formulations for migraines with the addition of prokinetic drugs could be beneficial for removing migraines and their associated disorders [28].

**Figure 2:**
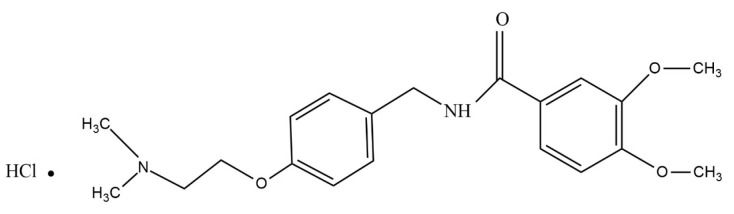
Chemical structure of ITHC.

Itopride hydrochloride (ITHC) is a novel benzamide gastroprokinetic derivative (shown in Figure 2) [29,30,31].

The drug has cooperative effects on both dopamine D2 receptor antagonistic and anticholinesterase (AnchE) activity [31]. Acetylcholine esterase (AchE) is an enzyme that hydrolyzes acetylcholine (ACh), which is being discharged from the terminating nerve and seems helpful in encouraging smooth muscle contraction by receptors, namely M3. Hydrolyzing activity causes their inactivation and decreases the motility of the gastric region and ultimately leads to gastric abnormalities. Alongside Ach, an occurrence with a considerable quantity dopamine in GIT also decreases the GIT motility, which ultimately decreases the intra-gastric and reduces lower esophageal sphincter pressure. The said results are due to the decrease in Ach release by myenteric motor neurons that were operated through a D_2_ subtype of dopamine receptors. ITHC works as an antagonist to the D_2_ dopamine receptor, ultimately reducing the repressive effects on Ach liberation. In turn, they also increase the intensity of Ach and depress the AchE concentrations, which turn in an enlarged concentration of Ach. This in return ultimately enhances gastroduodenal coordination, increases the pressure at the inferior sphincter of the esophageal, and increases stomach motility, causing gastric emptying [31,32,33]. The distinctiveness property of the ITHC mechanism of action is different from other pro-kinetic agents [31].

It is a member of the Biopharmaceutics Class System (BCS) class I drug [34,35], due to which it has a higher soluble capacity (>1000 mg/mL) and penetrability (cLog P¼2.65). The maximum plasma intensity was reached within 30 min after the oral administration of the active moiety. The oral bioavailability of the drug was not affected by food but could hinder its absorption rate, which will be delayed due to food. The drug was excreted through the kidneys in an unchanged form. It has a biological half-life of approximately 6 h [29,31]. The ITHC molecule breaks down into components, such as N-oxide, a metabolite, as a result of the oxidation of the tertiary amine N-dimethyl grouping due to the existence of flavin monooxygenase (FMO), a liver enzyme. The drug was excreted through the kidneys in an unchanged form. It has a biological half-life of approximately 6 h [29,31]. Due to its short half-life, it was a better candidate to be used in modified-release dosage forms to release the medication for an extended period and maintain the plasma drug level within the body. ITHC has no notable side effects on the cardiovascular and CNS. In addition, no teratogenic effect was found, which shows its abnormality in animals [31].

ITHC was intended for the management of functional dyspepsia and other gastrointestinal ailments [30], such as the feeling of gastric fullness, anorexia, nausea, vomiting, chronic gastritis, and upper adnominal pain [36]. The drug improves gastric motility disorders [37], promotes the emptying of gastric contents, comforts acid reflux and dyspepsia symptoms [38], and has an anti-emetic effect [39].

Since 1990, there appeared a 0.6% annual and a 14.6% total rise in migraineurs reported in Pakistan. Due to non-adherence to oral therapy and patient non-compliance, it was found that the condition of migraines worsens. The annual cost of a migraineur was estimated to be 144.8 dollars. Different classes of drugs were also prescribed for migraines, which ultimately increased the patient cost [40]. Gastroparesis was one of the major concerns to have occurred during the migraine attack. In the US, it was estimated that its prevalence increases up to 24.2 in 100,000 persons [41]. Therefore, to relieve the discomfort, nausea, and vomiting conditions that occurred due to gastroparesis, prokinetic agents were used [42], out of which ITHC is the best option. Therefore, the main purpose of the project was to develop a dosage form that encompasses both migraines and its subsidiary disorder gastropareses. To fulfill this, mixed buccal films of both active ingredients were prepared. They appeared to be beneficial and cost-effective.

### Advantages of Buccal Films

Buccal films exhibit the following advantages:

- Due to first-pass avoidance, the presence of high blood supply, and administration, oral mucosal ease is a striking route for the administration of medications.
- As buccal films remain intact for a longer period, they exhibit a higher release and availability of drugs compared to other routes, such as suspensions or solutions.
- The release pattern in films depends on their mode of use or formulation design. These can be used for both local and systemic effects. They can be prepared in one or more layers as well as in a modified release format.
- They increase patient compliance due to their small size, flexibility, and increased absorption time [43].

## 2. Results

The current studies were aimed to formulate buccal films for the co-administration of EHBR and ITHC for migraines and its associated symptoms, i.e., nausea and vomiting due to gastric stasis. The findings of the studies have advocated the successful accomplishment of film preparation. Out of 17 different trials, one formulation, having 650 mg of polymer, 130% of plasticizer, and 19.5% (F13) of surfactants, has been considered as optimum because of its considerable characteristics. Films were formulated using a central composite rotatable design (CCRD) model. The optimized formulation was further processed for various in vitro and in vivo evaluations.

### 2.1. Characterization of EHBR–ITHC Buccal Films

The outcomes of the selected formulation for different parameters were satisfactory, as pH was well within the desired range and the film was of a uniform thickness, having prompt disintegration and immediate dissolution. Furthermore, the FE and weight variations of the films were negligible, which leads to good content uniformity (Table 1).

### 2.2. In Vitro Dissolution and Ex Vivo Permeation Studies

**Figure 3:**
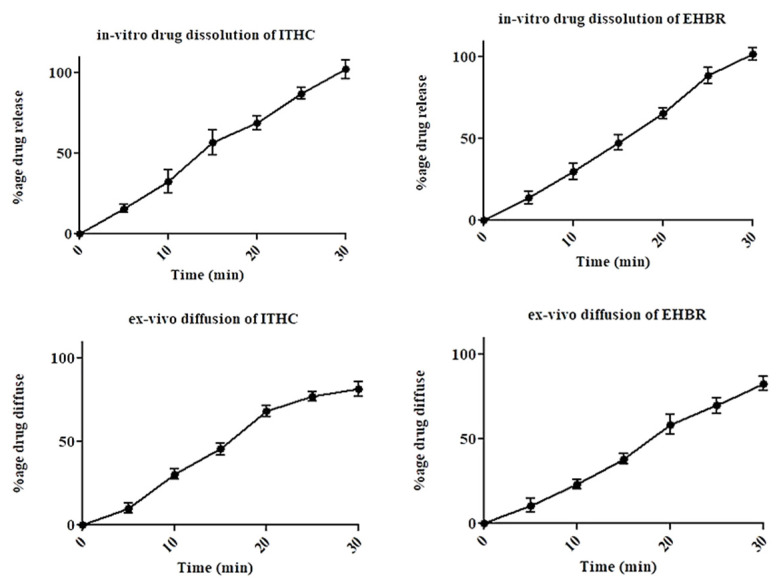
Representation of in vitro dissolution and ex vivo permeation of the drug from EHBR–ITHC buccal films.

Dissolution studies as well as ex vivo permeation studies have shown the rapid onset of dissolution and permeation of the drugs. Within 30 min of the studies, 100% of the drug has been released, and more than 80% of the drug is permeated across the buccal mucosa. The rapid release and permeation of the drug might be exhibited by the films due to some contributing factors, such as greater surface area, less thickness, and rapid disintegration. Figure 3, illustrated that, in the case of in vitro drug dissolution, a drug release of 101.8 ± 0.96 of ITHC and 100.21 ± 1.2% of EHBR was observed. A drug permeation of 80.8 ± 1.1% in the case of ITHC and 82.21 ± 0.97% of EHBR has been observed during ex vivo evaluations from their prepared films.

### 2.3. Scanning Electron Microscopy (SEM) of EHBR–ITHC Buccal Films

**Figure 4:**
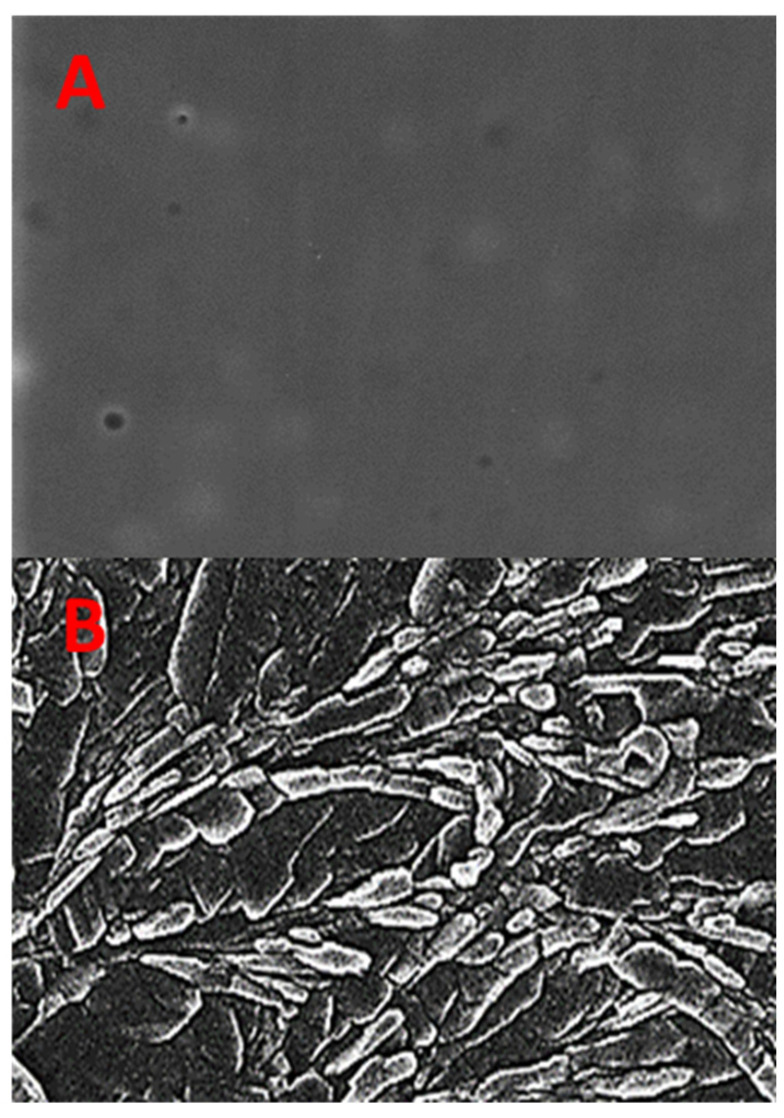
Scanning Electron Microscopy (SEM) image of (**A**) image of film and (**B**) image of a mixture of drugs.

The prepared films, i.e., with and without drugs, have been observed for surface morphology under SEM at 1000× magnification. The outcomes have revealed that the drug, polymer, and other excipients were thoroughly mixed, as none of the components has been identified as an individual entity (Figure 4A; image of film B; image of a mixture of drugs).

### 2.4. X-ray Diffractometer (XRD) of EHBR–ITHC Buccal Films

**Figure 5:**
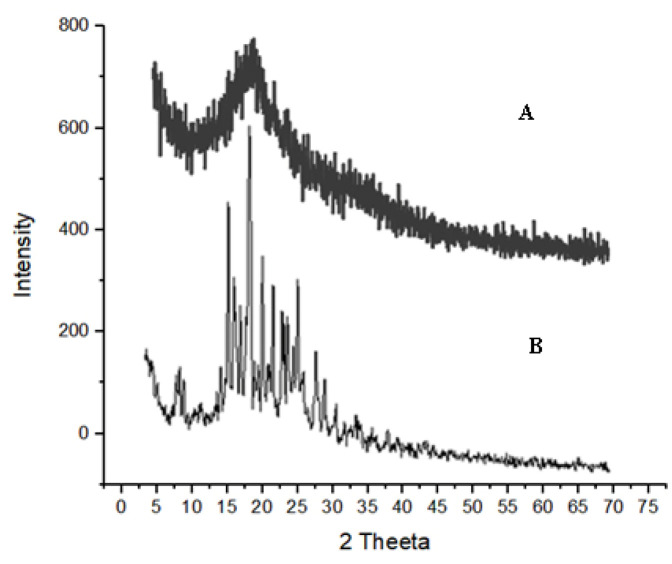
X-ray Diffractometer (XRD) image of combined films. (**A**) Prepared film of ITHC and EHBR co-loaded drugs. (**B**) Mixture of ITHC and EHBR drugs.

It was evident from the obtained XRD image of combined drugs, as shown in Figure 5A, that the drugs showed sharp peaks 2*θ* at 18°, 20°, 22°, and 25°, which illustrates the crystalline nature of the drugs. Followed by film formulation using their combination as represented in Figure 5B, there appeared no sharp peaks, which proves that the formulation possibly improves dissolution properties.

### 2.5. In Vivo Pharmacokinetic Analysis

Regarding the estimation and comparison of the extent of pharmacokinetic parameters, HPLC was used. The limit of detection (LOD) represents analyte concentration while the limit of quantification (LOQ) yields the noise-to-signal ratio [44]. In the case of EHBR, LOD and LOQ were 3.56 and 10.79 µg/mL while for ITHC, 1.5 and 4.6 µg/mL were observed for LOD and LOQ, respectively [45].

The in vivo performance of buccal films was compared with the oral dispersion of the pure drugs, followed by a statistical analysis. The one-way ANOVA was calculated using a PK solver. Furthermore, GraphPad Prism version 12 was used for the analysis of Bonferroni’s multiple comparisons.

The oral films of EHBR, ITHC, and their combination were prepared, and then, the pharmacokinetic parameters were compared. Following that, ITHC in combination with EHBR films were then analyzed with their dispersions. The results depict that the films represent a lower degree of t_1/2_ (h) in ITHC film 4.86 ± 0.02, EHBR film 4.70 ± 0.11, ITHC oral solution 5.01 ± 0.008, and EHBR oral solution 5.21 ± 0.009 when compared to their respective solutions. Similarly, regarding the C_max_, higher values were identified in the case of EHBR and ITHC films, which were 119 ± 0.14 and 130 ± 0.15, respectively. These results showed that prepared buccal films showed an effect and promote the effect when compared with oral dispersion for immediate drug release. One of the possible reasons for the immediate results in the case of films was because EHBR was lipophilic and its dispersion was prepared, which could decrease its efficiency as compared to formulated buccal films. Oral solution/dispersion has to pass through first-pass metabolism, whereas films bypass that metabolic mechanism and, ultimately, increase their immediate response.

**Figure 6:**
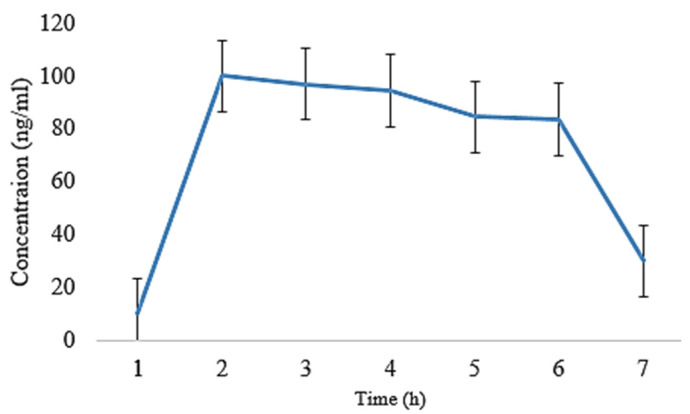
Plasma concentration of EHBR co-loaded film with ITHC after its oral administration to rabbits.

**Figure 7:**
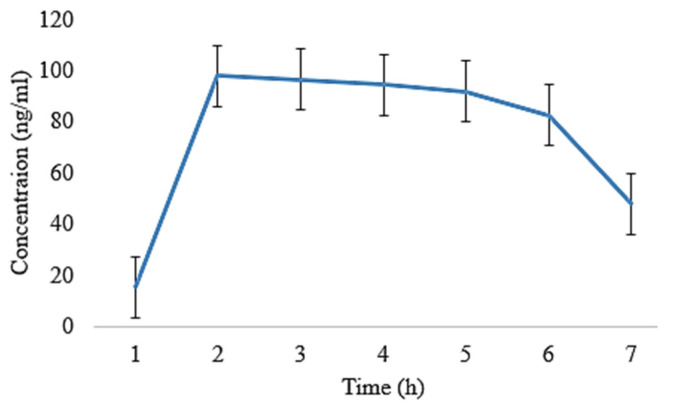
Plasma concentration of ITHC co-loaded film with EHBR after its oral administration to rabbits.

**Figure 8:**
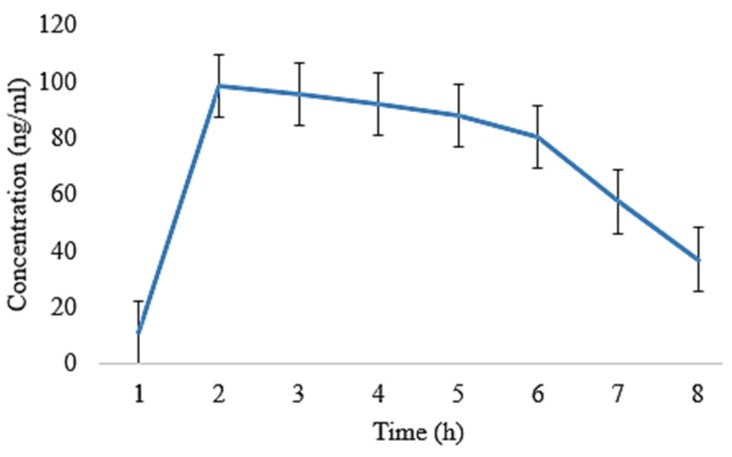
Plasma concentration of EHBR standard dispersion co-loaded with ITHC after its oral administration to rabbits.

**Figure 9:**
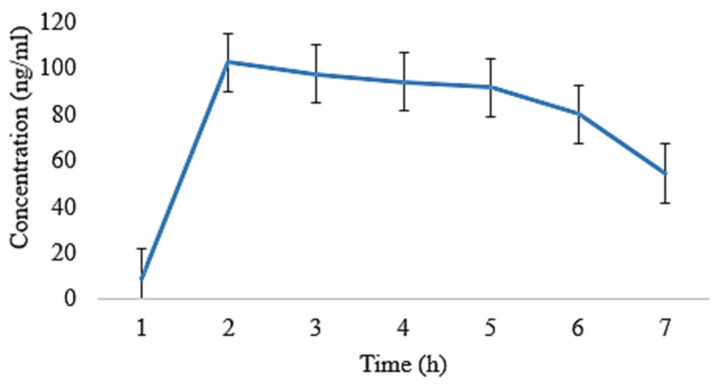
Plasma concentration of ITHC standard solution co-loaded with EHBR after its oral administration to rabbits.

It was obvious from Figure 6 and Figure 7 that the plasma level was observed to be higher in the film as compared to the oral solution (Figure 8 and Figure 9). In both of the cases, absorption was high, and it could possibly be due to ITHC belonging to the BCS class I drug, having high solubility and permeation.

All of these results were evident of an immediate release of the drug from the prepared films, which ultimately improved the bioavailability of the drug. Possible reasons for the higher drug release could be attributed to an increased surface area, decreased film thickness, and a high solubility profile of films due to the presence of glycerol and tween 80. Both of these excipients increase the film dissolution and eventually increase the solubility of the drug [46,47].

### 2.6. Statistical Analysis

For the comparative evaluation of formulated films and their standard solution, a statistical analysis was performed. First of all, a one-way ANOVA followed by Bonferroni’s multiple comparison test was applied using Graph Pad Prism version 12.

All of the parameters, such as t_1/2_, C_max_, and AUC, were significant, as shown in Table 2.

### 2.7. Histopathological Evaluation of Formulated Films

**Figure 10:**
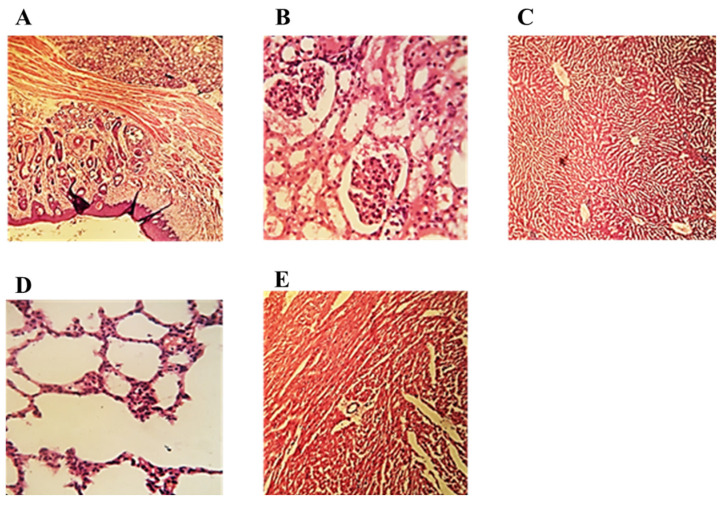
Histopathological slides of (**A**) buccal mucosa, (**B**) kidney tissues, (**C**) liver tissues, (**D**) lung tissues, (**E**) heart tissues.

The optimized buccal film was then investigated under a microscope for any histopathological change. For this evaluation, the buccal mucosa, kidney, heart, lung, and liver were visualized under the microscope. Figure 10 depicts that there appeared no damage or change in the cellular membrane. No cell necrosis was observed.

#### Kinetic Modeling

Kinetic models were useful to find out the release pattern of the drug using DD Solver software (Add-In MS Excel Microsoft Corporation USA, 2016). The formulated films were analyzed for different kinetic models, including zero-order model, first-order model, the Korsmeyer model, the Hixson–Crowell model, and the Higuchi model, and the value of *n* was tabulated in Table 3 for EHBR and Table 4 for ITHC formulated films.

It is observed from Table 3 that all the films formulated from EHBR have a best-fit model, which is the Korsmeyer–Peppas model, and the value of *n* lies between 0.383 to 0.792. Formulations F3, F5, F13, F15, and F16 follow the Hixson–Crowell model, and only one formulation, F10, follows the first-order model.

Films formulated by using ITHC showed that formulations F,1,2,7,8,9,13,14,16, and 16 have the Korsmeyer–Peppas model as the best-fit model. While film formulation F 3,5,10,11,12,15 follows the first-order model, as shown in Table 4.

## 3. Discussion

Formulated films were transparent and evenly thick with no signs of drug or polymer deposition. Rao et al. formulated buccal films of zolmitriptan, and it was evident from the work that the weight of the films was directly proportional to the number of excipients and drugs used [48,49]. Similar outcomes were observed in the current work, as the concentrations of the contents of the film also increase the weight of formulated films. However, the weight variation between strips of the same formulations was negligible, confirming the suitability of the process. This might be the reason that films have shown uniform drug contents as well [49].

The presence of moisture contents is useful to maintain the integrity and flexibility of the films [50]. Several previous works showed that the presence of surfactants reduces the interfacial tension within the formulation and increases the water retention capacity. The results showed that, as the concentration of surfactants (tween 80) increases, the moisture contents in the formulated films also increase [51]. The use of surfactant as well as plasticizer contributed to the retention of the moisture in the prepared films [52].

Both glycerol and PEG were reported as effective plasticizers and had proven to be factors that alter the mechanical properties of the films [52,53]. The outcomes of the current studies have strengthened the claim, as the % elongation at the break (%EB) of the films was possibly increased with the addition of plasticizers [54]. Ali Vijendar et al., researched to observe the impact of polymer and plasticizer on the %EB of films. The work states that elastic films were observed as the value of %EB increases and vice versa for brittle films [55]. Formulated films must have retained an optimum concentration of plasticity to make them feasible during administration, packaging, and transportation.

FE is also considered an important parameter for the evaluation of the mechanical properties of the films. The literature has suggested that films that represent an FE value greater than 200 times were considered to have a satisfactory elasticity property [56]. From the results, it was also observed that the increased concentration of HPMC could result in a decrease in the FE. On the other hand, an increased concentration of surfactant and plasticizer could increase the flexibility of formulated films. The addition of plasticizers structurally relaxes the hydrogen bonds, existing amongst the chains of polymer, and leads to an increase in the FE of films.

Permeation enhancers were used as buccal mucosa hinders the diffusion of agents across it. The permeation of the drugs was increased by the different mechanisms, which includes the alteration in membrane structure and rheology, its fluidity, and the extraction of the lipid layer. Hydration improves the permeability of different agents across the mucosa, and glycerol resolves the problem as it hydrates the mucosal membranes [57]. Different types of surfactants are present, out of which non-ionic surfactants were always preferred due to their non-toxicity and biocompatibility [58]. A common example of a non-ionic surfactant is tween 80. Tween 80 can easily be used for injectable, transdermal, oral, and mucosal formulations [59]. Tween 80 improves the permeability of pharmaceutical agents through the skin. It involves three main mechanisms. First, they originate the adsorption of molecules at the site of the skin and increase the force of molecules for permeation. Second, they reduce the *Stratum corneum* properties as a barrier. Third, they increase the drug permeation by modifying the skin layer, which causes a loss within the cell lipid layer [60].

Drug release profiling of all formulations showed that the films follow Korsmeyer–Peppas as the best-fit model. Other models, which include Hixson–Crowell and first-order models, were also followed by some formulations. Higher values of the Hixson–Crowell model advise us that the film was wholly dampened with media and releases of the drug [61]. Similarly, higher first-order values suggest that the release of the drug from formulated films depends upon their initial concentration. The value of *n* less than 0.5 indicates a Fickian model, and the release of the drug follows a diffusion mechanism, which states that the solvent transportation rate or diffusion was far larger than the development of polymeric chain leisure. Moreover, if the value of *n* is calculated greater than 0.5 but less than 0.89, it follows a non-Fickian or anomalous type of transport mechanism. The swelling and diffusion mechanism was favored for drug release [62]. The value of *n* greater than 1 meant that a formulation following super case II transport is recommended, as similar studies by Mane, PP et al., showed, where buccal films were developed using Polymer (HPMC) and Plasticizer (Propylene glycol) and the developed films followed super case II transport [63]. The super case II transport system confirms the drug release by both the diffusion and relaxation of the polymer chain [64].

## 4. Materials and Methods

ITHC (having a purity level of 99.66%) and EHBR (having a purity level of 101.0%) were gifted by CCL Laboratories, PVT, LTD, Lahore, Pakistan. HPMC E5 having a molar mass of 324.28 was gifted by Highnoon Laboratories, PVT, LTD, Lahore, Pakistan. Tween 80, dihydrogen potassium phosphate, sodium hydroxide, ethanol, and glycerol were purchased from Fisher Chemical, Loughborough, UK. The distilled water was taken from the Pharmacy Research Lab Faculty of Pharmaceutical Sciences, University of Central Punjab, Lahore, Pakistan.

### 4.1. Preparation of EHBR and ITHC Loaded Buccal Films

One of the simple and easy methods, i.e., solvent evaporation technique, has been employed for the development of buccal films of both drugs [65]. For ease and accuracy in preparation, ethanolic solutions of both drugs and aqueous solutions of individual excipients were prepared separately using a magnetic stirrer (79-1, Jiangsu Jinyi Instrument Technology Company Limited, Jintan, China). The concentrations of the solutions were as follows: 5% (*w*/*v*) aqueous solution of tween 80, 4% (*w*/*v*) aqueous solution HPMC E5, and 3% (*w*/*v*) of glycerol. The required volumes of all the individual ingredients were taken and mixed according to the formulation design (Table 5). All the formulations have been prepared and evaluated, and considering the outcomes of the optimized formulation (OF) are more considerable, formulation has been processed for further evaluations and in vivo pharmacokinetics. First, the polymeric solution was subjected to stirring using a magnetic stirrer, followed by the addition of tween 80, glycerol, and the drugs, one by one till the homogenous mixture was formed [54,66,67]. The prepared solution was covered using aluminum foil to prevent evaporation. The final mixture was then poured into Petri dishes and left to dry at 40 °C for 24 h in the oven. Afterward, the prepared films were peeled off and kept in aluminium foil for further use [67].

### 4.2. Evaluation of Buccal Films

#### Disintegration Time (DT) and Total Dissolving Time (TDT)

It is a function of film because the DT of the film varies with its composition. Characteristically, films disintegrate within 5 to 30 s [68,69]. The most common method for determining DT of the film is the petri dish method. For determination of DT through this method, the films were cut into the dimension of 2 × 2 cm^2^ and placed in 10 mL of distilled water, which was poured into a petri dish and slightly shaken periodically to determine the DT. The time by which the film started breaking was termed DT. The time by which the film completely dissolved in the water was known as total dissolving time (TDT). For better results, the procedure was repeated thrice, and the mean of the time was recorded to determine the DT and TDT of the films.

### 4.3. Surface pH

The pH of the buccal cavity lies within the range of 5.5 to 7.4. These films were designed to be placed within the buccal cavity, so their pH must lie in the pH range of the oral cavity, as the acidic or basic pH may irritate buccal mucosa. Formulated films were selected randomly for the evaluation of pH (Artesyn Embedded Technologies, Model AD2412N3L, Boca Raton, FL, USA). The films were divided into four equal portions of 2 × 2 cm^2^ and then moistened with water, followed by touching the tip of the pH electrode carefully to the moistened surface of the film. The electrode was kept in the same position till the constant value appeared. The same procedure was repeated thrice, and the mean has been calculated.

### 4.4. Moisture Content (MC)

For the evaluation of moisture loss and the level of moisture contents, MC is considered an important parameter. The MC is also useful to maintain the flexibility of the film. The films were divided into four equal parts and then placed in the oven at 50 °C until the constant weight was achieved. The following, Equation (1), was used to calculate the MC [70,71].

% moisture content (MC) = [Wo − Wd/Wo × 100](1)

where Wo represents the initial weight of the film and Wd is the dried weight.

### 4.5. Thickness

To ensure the uniform distribution of drugs and excipients within the formulated film, the films should be of uniform thickness. The thickness was measured using a micrometer that was employed to determine the thickness from different regions of the same strip in triplicate, and their mean was recorded.

### 4.6. Folding Endurance (FE)

One of the parameters to evaluate the mechanical properties of films is FE. It is considered an important parameter to assess the mechanical strength of the formulated films, which prevent the breakage before its administration. The strength of the films was evaluated by folding the films at 180° angle from the same point until they cracked. The higher the FE, the higher the mechanical strength.

### 4.7. Content Uniformity

The prepared films were cut into 2 × 2 cm^2^ portions from different areas, and the strips were dissolved in a buffer of pH 6.8. The sample from the solution was taken and subjected to analysis using a UV–Vis spectrophotometer (Halo DB-20 UV, Progen Scientific, London, UK) [72].

### 4.8. Scanning Electron Microscopy (SEM)

The *scanning* morphology of films is important to observe the surface texture and smoothness. The SEM (EVO LS-10 ZEISS Germany, Carl Zeiss NTS, Oberkochen, Germany) was used to observe the morphological characteristics at the micro level.

### 4.9. X-ray Diffraction (XRD)

The XRD is an important parameter that has been used to observe the crystalline or amorphous nature of the drug and the impact of formulation variables on the said characteristics of the drug. The X-ray diffractometer (JDX-3532 JEOL, Tokyo, Japan) has been used to study the XRD of pure drugs and prepared formulations [73].

### 4.10. In Vitro Dissolution Studies

The rate of dissolution of formulated films was studied using dissolution apparatus II (USP type-II paddle apparatus, BK-RC series, Bio base, Jinan, China). The instrumental conditions were set as follows: the paddles of the apparatus were rotated at 50 rpm, and the temperature was maintained at 37.0 °C ± 0.5 °C. The vessels were filled with 250 mL of prepared simulated artificial saliva that had a pH of 5.7 as dissolution media. A sample volume of 5 mL was withdrawn after 0, 5, 10, 15, 20, 25, and 30 min, filtered using 0.45 μm syringe filters (Whatman, Sigma Aldrich, Baltimore, MD, USA) and analyses using the prepared buffer, and analyzed in UV spectrophotometer at λ_max_ 250 nm for ITHC and EHBR at 220 nm wavelengths [74].

### 4.11. Ex Vivo Permeation Studies

The permeation studies were conducted using the Franz diffusion cell. Freshly excised goat buccal mucosa was taken from the slaughter house of the local market and preserved in normal saline for further use. The buccal mucosa was placed between the donor and the receiver compartments of the Franz diffusion cell. These cells have a diffusional area of 1.76 cm^2^ using simulated artificial saliva-based buffer that has pH 5.7 as media. The receiver compartment was filled with the media having a volume of 5 mL. The buccal mucosa was mounted over the receiver compartment, followed by the placement of the film over the mucosa. After that, donor compartment was placed over it and clumped together with the help of clumps. The entire assembly was maintained at 37.0 °C ± 0.5 °C. A sample of 1 mL from the receiver compartment was withdrawn every time and replaced with buffer that was maintained at the same temperature. Samples were withdrawn at 0, 5, 10, 15, 20, 25, and 30 min and analyzed using UV spectrophotometer (Halo DB-20 UV, Progen Scientific, London, UK) at 220 and 250 nm for EHBR and ITHC, respectively.

### 4.12. In Vivo Pharmacokinetic Study

The applicability of prepared formulations could be assessed with pharmacokinetics using any suitable model. In current studies, the films have been subjected to pharmacokinetic evaluation using albino rabbits [75,76,77]. The films were placed in the oral cavity against the buccal mucosa of the partially anesthetized animal. The blood samples (Iml) from the marginal ear veins were derived after different time intervals and subjected to drug extraction with liquid–liquid extraction technique. Initially, the blood sample was centrifuged at 6000 rpm for 30 min to remove the blood cells. The supernatant was mixed with 3 mL of ethanol, and the same procedure was repeated. The supernatant was separated again, mixed with 3 mL of diethyl ether, and subjected to centrifugation under the same conditions. The precipitants were separated, and the supernatant was placed in a water bath at 50 °C to evaporate the solvent, leaving behind the dried residue of the drug. The dried sample was re-constituted with buffer medium, and 20 µL of the sample were injected into HPLC to observe different pharmacokinetic parameters, including area under the curve (AUC), peak plasma concentration (C_max_), time to reach maximum plasma concentration (T_max_), and half-life of the drug (t_1/2_). The trapezoidal rule was used to ascertain the area under the plasma time concentration curve from 0 to 24 (AUC_0–24_) and from 0 to infinity (AUC_0–∞_ ng h/mL). The latter can be determined from AUC_0–24_ as; AUC_0–∞_ = AUC_0–24_ + C*/k using mathematical expression (2):

(2) $$AUC=\frac{1}{2}(C_{1}+C_{2})(t_{2}-t_{2}),$$

where t_1_ is the initial time, t_2_ is the final time, C_1_ is the initial concentration, and C_2_ is the final concentration of the drug.

On the other hand, C_max_ has been calculated from Equation (3) as follows:

(3) $$C_{max}=\frac{FX_{0}}{V_{d}}\times {exp}^{-ke}*t_{max}$$

While F was the dose fraction, K_e_ was the rate constant for elimination, and the volume of distribution was represented by V_d_. Similarly, the T_max_ was determined using Equation (4):

(4) $$t_{max}=2.303(\log K_{a}/K_{e})(K_{a}-K_{e}),$$

where K_e_ was the elimination rate constant and K_a_ was the absorption rate constant.

The half-life (t_1/2_) is the time by which half of the drug was excreted out of the body and was calculated using Equation (5):

(5) $$t_{1/2}=0.693/k$$

The variable k was represented as a rate constant [78].

#### Chromatographic Condition

The mobile phase was prepared using buffer and acetonitrile in 70:30 ratios, with an adjusted pH of 4.6 using orthophosphoric acid. The C 18 column having dimensions of 250 mm × 4.6 mm and particle size of 5 μm was used for HPLC analysis at 25 °C, where the flow rate was 1 mL/min with the injection volume of 10 µL.

### 4.13. Statistical Analysis

Graph pad prism ver. 12 was used for the statistical analysis of data. For the comparison of more than two groups, ANOVA with Bonferroni’s multiple comparison tests was used; meanwhile, for the comparison of two groups, unpaired *t*-test was used. The data were interpreted at a 95% confidence interval with their standard deviation (SD) calculated as mentioned. The value of *p* indicated the value of significance where *p* < 0.05 indicated that data were significant (*), *p* < 0.01 represented very significant data (**), and *p* < 0.0001 showed extremely significant data (*** or ****) [79].

### 4.14. Histopathological Evaluation of Formulated Films

Histopathological examinations were conducted to observe the compatibility and safety of the prepared films. The prepared films were placed on the buccal mucosa of the rabbits, and after the complete absorption of the films, the mucosa was cut off and immediately placed in a 10% solution of formalin. As both drugs reached the systemic circulation of the rabbit, the major organs, which include the kidney, liver, lungs, and heart, were also evaluated. For the preparation of histological slides, the slides were stained with hematoxylin and eosin. Finally, the prepared slides were assessed under a light microscope to detect any histopathological variations [80].

### 4.15. Kinetic Modeling

Kinetic models were useful to find out the release pattern of the drug using DD Solver software (Add-In MS Excel Microsoft Corporation USA, 2016). The prepared films were analyzed and found for different kinetic models, including first-order model, zero-order model, Korsmeyer model, Hixson–Crowell model, and Higuchi model, and the value of *n* was tabulated.

## 5. Conclusions

All of the above-mentioned objectives of the current work were achieved. Mucoadhesive-loaded films of EHBR and ITHC were successfully fabricated using HPMC E 5 as a film-forming agent. HPMC reportedly acts as a brilliant film former and IR carrier. Outstanding results demonstrate that the combination of ITHC and EHBR with HPMC E 5 proved to be successful and effective for the preparation of two active ingredients at one time. Fabricated films not only enhance patient compliance in terms of easy administration, but also proved to be beneficial regarding the deliverance of two drugs at the same time. The results of the in vitro, ex vivo, and in vivo techniques portray that the combination therapy of ITHC and EHBR in migraines and the associated syndromes appeared to be beneficial.

## Figures and Tables

**Table 1:** Outcomes of selected formulation.

pH	Thickness (mm)	DT (s)	TDT (s)	Weight Variation (mg)	MC (%)	FE	Content Uniformity	
							EHBR (%)	ITHC (%)
6.5 ± 0.02	0.23 ± 0.1	13 ± 1	42.6 ± 0.75	0.035 ± 0.05	45 ± 0.78	249 ± 1	101 ± 0.1	97 ± 0.3

**Table 2:** Pharmacokinetics of ITHC and EHBR co-loaded film after its oral administration compared with standard oral dispersion/solution, which contains the drug in the quantity of 15 mg/kg and 1 mg/kg dose.

Parameters	ITHC–EHBR Buccal Films		ITHC–EHBR Solution	
	ITHC	EHBR	ITHC	EHBR
t_1/2_ (h)	4.86 ± 0.02	4.70 ± 0.11	5.01 ± 0.008	5.21 ± 0.009
t_max_ (h)	1 ± 0.01	1 ± 0.05	1 ± 0.02	1 ± 0.03
C_max_ (ng/mL)	130 ± 0.15	119 ± 0.14	96 ± 0.11	90.5 ± 0.07
Clast_obs/C_max_	0.32 ± 0.01	0.43 ± 0.05	0.460 ± 0.01	0.46 ± 0.03
AUC_0–t_ (ng/mL·h)	693 ± 1.01	650 ± 1.20	648.5 ± 0.29	639.5 ± 0.55
AUC_0–inf_obs_ (ng/mL·h)	987.76 ± 1.06	881.76 ± 1.19	993.03 ± 1.21	984.03 ± 1.11
AUC_0–t/0–inf_obs_	0.701 ± 0.11	0.737 ± 0.14	0.65 ± 0.10	0.649 ± 0.13
AUMC_0–inf_obs_ (ng/mL·h^2^)	6966.73 ± 1.12	5772.72 ± 1.08	7749.29 ± 1.23	7747.79 ± 1.11
MRT_0–inf_obs_ (h)	7.05 ± 0.16	6.30 ± 0.35	7.80 ± 0.16	7.87 ± 0.13

**Table 3:** Kinetic modeling of Drug Diffusion (DD) from EHBR formulations.

Formulation No	Zero-Order	First-Order	Highuci Model	Hixson–Crowell Model	Korsmeyer–Peppas Model	Value of *n*	Best-Fit Model
1	0.5169	0.9646	0.9723	0.9157	0.9733	0.503	Korsmeyer–Peppas model
2	0.5067	0.9616	0.9590	0.9101	0.9690	0.500	Korsmeyer–Peppas model
3	0.5605	0.9748	0.9670	0.9870	0.9680	0.519	Hixson–Crowell model
4	0.5597	0.8696	0.9475	0.8148	0.9485	0.520	Korsmeyer–Peppas model
5	0.5605	0.9748	0.9670	0.9870	0.9680	0.519	Hixson–Crowell model
6	0.9498	0.9527	0.8907	0.9787	0.9878	0.783	Korsmeyer–Peppas model
7	0.6912	0.9064	0.9555	0.8533	0.9659	0.568	Korsmeyer–Peppas model
8	0.9478	0.9608	0.8819	0.9720	0.9813	0.792	Korsmeyer–Peppas model
9	0.2271	0.5515	0.8997	0.3480	0.9664	0.383	Korsmeyer–Peppas model
10	0.1243	0.9728	0.9035	0.9556	0.9539	0.395	First-order
11	0.1638	0.6226	0.8813	0.4270	0.9378	0.389	Korsmeyer–Peppas model
12	0.7101	0.8939	0.9566	0.8459	0.9694	0.576	Korsmeyer–Peppas model
13	0.9183	0.9509	0.9044	0.9711	0.9799	0.736	Hixson–Crowell model
14	0.8471	0.9666	0.9504	0.9416	0.9890	0.648	Korsmeyer–Peppas model
15	0.5605	0.9748	0.9670	0.9870	0.9680	0.519	Hixson–Crowell model
16	0.1121	0.9942	0.9161	0.9806	0.9378	0.425	Hixson–Crowell model
17	0.7591	0.9240	0.9637	0.8825	0.9826	0.595	Korsmeyer–Peppas model

**Table 4:** Kinetic modeling of Drug Diffusion (DD) from ITHC formulations.

Formulation No	Zero-Order	First-Order	Highuci Model	Hixson–Crowell Model	Korsmeyer–Peppas Model	Value of *n*	Best-Fit Model
1	0.9540	0.8877	0.7325	0.9186	0.9585	1.101	Korsmeyer–Peppas model
2	0.8067	0.8844	0.9222	0.9650	0.9872	0.641	Korsmeyer–Peppas model
3	0.7041	0.9825	0.9479	0.9601	0.9602	0.575	First-order
4	0.9011	0.9613	0.8512	0.9650	0.9424	0.774	Hixson–Crowell model
5	0.7041	0.9825	0.9479	0.9601	0.9602	0.575	First-order
6	0.7462	0.9655	0.9302	0.9808	0.9513	0.603	Hixson–Crowell model
7	0.9583	0.8873	0.7327	0.9192	0.9638	1.114	Korsmeyer–Peppas model
8	0.8635	0.9610	0.9162	0.9706	0.9882	0.682	Korsmeyer–Peppas model
9	0.0773	0.9324	0.8326	0.8553	0.9738	0.400	Korsmeyer–Peppas model
10	0.7004	0.9549	0.9049	0.9201	0.9214	0.591	First-order
11	0.7652	0.9462	0.8641	0.9401	0.8999	0.648	First-order
12	0.9217	0.9685	0.8588	0.9738	0.9574	0.789	First-order
13	0.9766	0.8681	0.7623	0.9145	0.9787	1.067	Korsmeyer–Peppas model
14	0.9799	0.9730	0.8133	0.9810	0.9814	0.948	Korsmeyer–Peppas model
15	0.7041	0.9825	0.9479	0.9601	0.9602	0.575	First-order
16	0.9941	0.9752	0.7975	0.9844	0.9943	1.022	Korsmeyer–Peppas model
17	0.9844	0.9430	0.8018	0.9677	0.9846	0.981	Korsmeyer–Peppas model

**Table 5:** Composition of ITHC and EHBR loaded buccal films having variable concentrations of polymer, plasticizer, and surfactant according to CCRD.

Trials	Polymer (mg)	Plasticizer (%)	Surfactant (%)
1	500.00	87.5	28.4
2	500.00	66.5	20
3	500.00	87.5	20
4	500.00	87.5	11.6
5	500.00	87.5	20
6	350.00	70	10.5
7	350.00	52.5	10.5
8	500.00	108.5	20
9	650.00	97.5	32.5
10	650.00	130	32.5
11	350.00	70	17.5
12	650.00	97.5	19.5
13	650.00	130	19.5
14	752.27	131.7	30.1
15	500.00	87.5	20
16	247.73	43.4	9.9
17	350.00	52.5	17.5

In all formulations a constant amount of drug ITHC (200 mg) and EHBR (80 mg) was used.

## Data Availability

Data is contained within the article.
